# Supplementary material for: Normative Modeling of Molecular-Enriched Functional Connectivity for Detecting Deviations from Healthy Brain Aging
Source: bioRxiv. 2026 Sep 29:2026.09.28.754989. Preprint. [Version 1] doi: 10.64898/2026.09.28.754989 (PMC13648635; doi:10.64898/2026.09.28.754989)
Supplement: Supplementary file 1 [file NIHPP2026.09.28.754989v1-supplement-1.pdf]

bioRxiv preprint doi: https://doi.org/10.64898/2026.09.28.754989; this version posted September 29, 2026. The copyright holder for this preprint (which was not certified by peer review) is the author/funder, who has granted bioRxiv a license to display the preprint in perpetuity. It is made available under a CC-BY 4.0 International license.

A Supplementary Materials

A.1 Image processing

A.1.1 MRI preprocessing and quality control

Healthy normative reference cohort. The raw T1w and rs-fMRI data from all seven normative reference datasets were processed using an identical workflow to minimize methodological variability across cohorts. The organization of imaging data followed the Brain Imaging Data Structure (BIDS) specification (Gorgolewski et al., 2016), and the data were processed using standardized, BIDS-compatible neuroimaging tools. The degree to which the BIDS specification was adhered to was determined by means of the BIDS Validator (v1.14.6) prior to image processing (Blair et al., 2024).

Quality control. The assessment of image quality was conducted by employing the MRIQC (v24.1.0) tool (Esteban et al., 2017), with each dataset and imaging modality undergoing independent evaluation. Given the substantial number of scans included in the healthy normative reference cohort, QC followed a semi-automated procedure combining quantitative image-quality metrics (IQMs) with targeted visual inspection. Group-level MRIQC reports were examined separately within each dataset, and scans with IQMs falling outside the inner Tukey fences, defined as values below $Q_1 - 1.5 \times \mathrm{IQR}$ or above $Q_3 + 1.5 \times \mathrm{IQR}$, were flagged for subsequent visual assessment (Tukey, 1977).

For T1w images, the quality assessment focused on signal-to-noise ratio (SNR), gray-to-white-matter contrast-to-noise ratio, and the entropy-focus criterion. For rs-fMRI data, temporal signal-to-noise ratio (tSNR), mean framewise displacement (FD), and the temporal derivative of the variance of the BOLD signal (DVARS) were considered. All scans that were flagged by the quantitative procedure were then subjected to a visual inspection. Images that exhibited substantial motion, ringing, cropping, or other evident acquisition artifacts were excluded from further processing.

Structural and functional preprocessing with fMRIPrep. Preprocessing of the structural and fMRI data was conducted using fMRIPrep (v23.2.2) (Esteban et al., 2019, 2020). For each participant, T1w images underwent intensity non-uniformity correction using N4 bias-field correction, implemented in Advanced Normalization Tools (ANTs) (v2.4.4) (Tustison et al., 2010). The skull stripping procedure was executed employing the Nipype implementation of `antsBrainExtraction.sh` which utilized the OASIS30ANTs template distributed through TemplateFlow (v23.1.0) (Ciric et al., 2022). The brain-extracted T1w image was segmented into CSF, WM, and gray matter (GM) using FAST from FSL

bioRxiv preprint doi: https://doi.org/10.64898/2026.09.28.754989; this version posted September 29, 2026. The copyright holder for this preprint (which was not certified by peer review) is the author/funder, who has granted bioRxiv a license to display the preprint in perpetuity. It is made available under a CC-BY 4.0 International license.

(v6.0.6.2) (Zhang et al., 2001). Cortical surface reconstruction was performed using `recon-all` from FreeSurfer (v7.3.2) (Fischl, 2012), and the initial brain mask was refined by reconciling the ANTs- and FreeSurfer-derived gray-matter segmentations using a Mindboggle-based procedure (Klein et al., 2017). Subsequently, the anatomical images were nonlinearly normalized to the MNI ICBM 152 nonlinear sixth-generation asymmetric template (MNI152NLin6Asym) at 2-mm isotropic resolution (Ciric et al., 2022; Evans et al., 2012). Nonlinear registration was performed using `antsRegistration` from ANTs (Avants et al., 2008, 2011).

For each rs-fMRI acquisition, a BOLD reference image was generated and head-motion parameters were estimated using `mcflirt` from FSL before temporal filtering (Jenkinson et al., 2012). Subsequent to this, slice-timing correction was performed, and susceptibility-distortion correction was applied when the required fieldmap information was available. The BOLD reference image was co-registered with the corresponding T1w image using FreeSurfer's boundary-based registration (`bbregister`) with six degrees of freedom (Greve & Fischl, 2009). A series of transformations were concatenated and applied in a single interpolation step using `nitransforms` with cubic B-spline interpolation. These transformations included head motion, BOLD-to-T1w, and T1w-to-MNI spatial transformations.

Framewise displacement (FD) and DVARS were retained as framewise motion-sensitive quality metrics (Power et al., 2012). For the present preprocessing workflow, volumes exceeding either FD $>$ 0.5 mm or standardized DVARS $>$ 1.5 were classified as motion-affected frames for subsequent interpolation. No nuisance regression or temporal filtering was applied within fMRIPrep.

Post-processing with XCP-D. The fMRIPrep derivatives were subsequently denoised using XCP-D (v0.7.4) (Mehta et al., 2024). Confound regression was executed in accordance with the `acompcor` nuisance-regression strategy, encompassing the six rigid-body motion parameters and their temporal derivatives, in conjunction with the ten leading anatomical CompCor (aCompCor) components derived from WM and CSF (Behzadi et al., 2007; Satterthwaite et al., 2013).

Frames that were identified as being affected by motion were reconstructed using cubic-spline interpolation to ensure the preservation of a temporally continuous BOLD signal for the purpose of subsequent REACT analysis. The BOLD time series and nuisance regressors underwent temporal high-pass filtering using a second-order Butterworth filter with a cutoff frequency of 0.01 Hz. Subsequently, the nuisance effects were eliminated through the implementation of linear regression with the specified confound set and cosine basis functions. This process followed established methodologies for the mitigation of motion-related artifacts in FC analyses (Ciric et al., 2018). Given that REACT necessitates continuous functional time series, the interpolated, non-censored residual BOLD images generated by XCP-D were preserved for

bioRxiv preprint doi: https://doi.org/10.64898/2026.09.28.754989; this version posted September 29, 2026. The copyright holder for this preprint (which was not certified by peer review) is the author/funder, who has granted bioRxiv a license to display the preprint in perpetuity. It is made available under a CC-BY 4.0 International license.

subsequent processing. No spatial smoothing was applied within XCP-D.

Spatial smoothing. Following XCP-D post-processing, the interpolated residual BOLD images were spatially smoothed using FSL (v6.0.6.2) with an isotropic Gaussian kernel of 6 mm FWHM, as described in Jenkinson et al. (2012) and Smith et al. (2004). The resulting denoised and spatially smoothed BOLD images constituted the functional input to the subsequent REACT analysis.

AMYPAD-PNHS cohort. The rs-fMRI data from the AMYPAD-PNHS cohort had previously undergone a standardized preprocessing workflow, as described by Arunachalam et al. (2026). The preprocessing of functional images was conducted using fMRIPrep (v23.0.1). In summary, the preprocessing stage entailed the estimation of a BOLD reference image, the extraction of head-motion parameters, and the implementation of slice-timing correction when applicable. Corrections for susceptibility-related distortions were implemented through the utilization of a fieldmap-less approach, which was based on the non-linear registration of the BOLD reference image to the corresponding T1w image. This registration was accomplished through the implementation of symmetric normalization in ANTs. Subsequent to this, the functional images were aligned to the T1w image using FreeSurfer's boundary-based registration and transformed with ANTs to MNI152NLin6Asym standard space at 2-mm isotropic resolution, matching the space used for the molecular templates and regional feature-generation workflow.

Thereafter, the initial non-steady-state volumes were removed following fMRIPrep preprocessing. The functional time series were spatially smoothed using a Gaussian kernel with a FWHM of 4 millimeters. The nuisance regression model incorporated six rigid-body head-motion parameters, along with the mean cerebrospinal-fluid and white-matter signals. Linear trends were removed, and the resulting time series were temporally band-pass filtered between 0.01 and 0.1 Hz. The resulting preprocessed BOLD images constituted the functional input to the molecular-enriched connectivity analyses performed in the present study.

From this stage onward, the AMYPAD-PNHS and healthy normative-reference cohorts underwent the same molecular-enriched feature-generation workflow. Specifically, REACT was applied to the AMYPAD-PNHS data using the same DAT, NET, and SERT molecular templates, analysis masks, and two-stage regression procedure described in Section A.1.2. The resulting subject-specific molecular-enriched FC maps were then parcellated using the same anatomical definitions and summarized using the same regional mean-extraction procedure described in Section A.1.2. This common downstream feature-definition strategy yielded nominally matched regional molecular-enriched functional-connectivity variables in the normative-reference and AMYPAD-PNHS cohorts, providing the feature correspondence required for

bioRxiv preprint doi: https://doi.org/10.64898/2026.09.28.754989; this version posted September 29, 2026. The copyright holder for this preprint (which was not certified by peer review) is the author/funder, who has granted bioRxiv a license to display the preprint in perpetuity. It is made available under a CC-BY 4.0 International license.

normative-model transfer while leaving upstream acquisition and preprocessing differences to be addressed by hierarchical adaptation and local calibration.

The use of independently established upstream preprocessing workflows was deliberate and formed part of the transferability evaluation. AMYPAD-PNHS raw data were not retrospectively reprocessed with the complete normative-reference preprocessing pipeline, and no explicit feature-level harmonization was applied before normative-model transfer. Instead, cross-cohort compatibility was imposed at the molecular-enriched feature-definition stage by applying the same REACT templates, analysis masks, cortical parcellation, and regional extraction procedure. Hierarchical transfer and local calibration were then used to test whether the pretrained NM could accommodate the target-domain distribution under this deployment-oriented scenario.

A.1.2 Molecular-enriched functional connectivity

Receptor-Enriched Analysis of functional Connectivity by Targets. Molecular-enriched FC was derived using REACT (Dipasquale et al., 2019). REACT integrates individual rs-fMRI BOLD fluctuations with population-level molecular density templates obtained using PET or SPECT.

The analysis comprised two consecutive multivariate general linear models (GLMs). In Stage 1, the DAT, NET, and SERT molecular-density templates were entered jointly in a single spatial design matrix and regressed against each participant's BOLD data. This yielded three subject-specific target-weighted time series while accounting for spatial covariance among the molecular templates. Stage 1 was restricted to the intersection of the common positive support of the three molecular templates, which were supplied together as a single four-dimensional atlas, and the gray-matter mask distributed with the REACT implementation. In Stage 2, the three Stage-1 time series were entered jointly into a temporal regression against the voxel-wise BOLD signal within the gray-matter analysis mask, yielding one subject-specific molecular-enriched FC map for each molecular target. Joint inclusion of molecular maps is important when templates share spatial variance, because omission of correlated molecular systems can alter target-specific attribution of BOLD variance (Lawn et al., 2023); previous multitemplate REACT work that included the present DAT, NET, and SERT templates evaluated this issue using spatial correlations and variance-inflation diagnostics (Lawn et al., 2024). The same joint design, masks, and template definitions were used for the normative-reference and AMYPAD-PNHS cohorts.

Molecular templates. Three monoaminergic transporter templates were included: the DAT, NET, and SERT. The DAT template was derived from $[^{123}\mathrm{I}]$-ioflupane SPECT data from 26 subjects with no

bioRxiv preprint doi: https://doi.org/10.64898/2026.09.28.754989; this version posted September 29, 2026. The copyright holder for this preprint (which was not certified by peer review) is the author/funder, who has granted bioRxiv a license to display the preprint in perpetuity. It is made available under a CC-BY 4.0 International license.

evidence of nigrostriatal degeneration (García-Gómez et al., 2013). The NET template was generated from $[^{11}\mathrm{C}]$MRB PET parametric maps acquired from 10 healthy individuals (Hesse et al., 2017). The SERT template was derived from the $[^{11}\mathrm{C}]$DASB PET component of a high-resolution serotonin atlas built from 210 healthy individuals aged 18–45 years (Beliveau et al., 2017). All templates were represented in MNI152NLin6Asym space at 2-mm isotropic resolution and scaled between 0 and 1.

Regional feature extraction. Subject-specific DAT-, NET-, and SERT-enriched FC maps were summarized using the DK cortical parcellation. For each molecular system, the mean molecular-enriched FC value was extracted from 34 DK regions in each hemisphere. These regions included the banks of the superior temporal sulcus, caudal anterior cingulate, caudal middle frontal, cuneus, entorhinal, fusiform, inferior parietal, inferior temporal, isthmus cingulate, lateral occipital, lateral orbitofrontal, lingual, medial orbitofrontal, middle temporal, parahippocampal, paracentral, pars opercularis, pars orbitalis, pars triangularis, pericalcarine, postcentral, posterior cingulate, precentral, precuneus, rostral anterior cingulate, rostral middle frontal, superior frontal, superior parietal, superior temporal, supramarginal, frontal pole, temporal pole, transverse temporal, and insula. This yielded 68 cortical measurements for each molecular system and 204 cortical molecular-system features in total (68 DAT, 68 NET, and 68 SERT). A separate normative model was estimated for each cortical molecular-system feature. For the AMYPAD-PNHS cohort, subject-specific FreeSurfer DK labels were those produced by the standard AMYPAD structural processing workflow, which uses FreeSurfer v7.1.1 for cortical reconstruction and parcellation. For regional extraction, subject-specific FreeSurfer DK labels were propagated from FreeSurfer native anatomical space to T1w space and then to MNI152NLin6Asym space by combining the subject-specific FreeSurfer-native-to-T1w registration with the fMRIPrep T1w-to-MNI152NLin6Asym transform. Label images were resampled with nearest-neighbor interpolation to preserve discrete parcel identities before regional averaging of the REACT maps.

A.2 Sensitivity analyses

Sensitivity analyses evaluated whether the principal findings depended on model-diagnostic filtering, deviation-score representation, downstream covariate adjustment, or RADB distributional handling. First, the principal analyses were repeated in the strict cross-batch diagnostic sensitivity domain containing only cortical features that satisfied both the normative-model and transfer-diagnostic criteria in all three AMYPAD acquisition batches. Regional multiplicity correction in this sensitivity analysis was applied across the retained diagnostic feature domain, while subject-level FDR correction remained across the four global/system-specific scopes.

bioRxiv preprint doi: https://doi.org/10.64898/2026.09.28.754989; this version posted September 29, 2026. The copyright holder for this preprint (which was not certified by peer review) is the author/funder, who has granted bioRxiv a license to display the preprint in perpetuity. It is made available under a CC-BY 4.0 International license.

Second, independent intermediate-versus-positive analyses were repeated using model-native Z^{raw}. Because this score can retain residual batch-specific location and scale differences, regional and RADB effects were estimated separately within each acquisition batch using HC3 regression adjusted for age and sex. Each batch-specific coefficient and SE was divided by the corresponding model residual SD to obtain a standardized effect, and standardized effects were combined across batches using inverse-variance fixed-effect meta-analysis. Cochran's Q and I^2 characterized between-batch heterogeneity. The same batch-stratified strategy was used for Z^{raw} continuous CL analyses. FDR correction followed the same 204-feature regional or four-scope subject-level families used in the corresponding atlas-complete primary analyses.

For Z^{raw} extreme-deviation burden, a universal $|Z| > 1.96$ threshold was not applied because Z^{raw} could retain batch-dependent scaling. Instead, feature- and batch-specific empirical 2.5th and 97.5th percentile limits were estimated from the corresponding local amyloid-negative reference participants, and Z^{raw} extreme-deviation burden was defined as the proportion of features falling outside these empirical local 95% limits.

RADB distributional sensitivity analyses flagged values beyond a 3×IQR outer fence within each global or molecular-system-specific scope; primary analyses retained these observations, while sensitivity models were repeated after their exclusion. RADB group analyses were also repeated after natural-log transformation.

A.2.1 Effects of alternative analytical specifications

Restricting the analyses to the 157 cortical features that satisfied both normative and transfer diagnostic criteria in all three AMYPAD batches yielded adjusted intermediate minus amyloid-positive RADB differences of 0.169 for the global scope, 0.153 for DAT, 0.086 for NET, and 0.164 for SERT; none survived FDR correction (global, DAT, and SERT $q = 0.063$; NET $q = 0.283$). The DAT extreme-deviation-burden difference was 0.029 (95% CI 0.004–0.054, $p = 0.022$, $q = 0.090$). Within this strict diagnostic sensitivity domain, no tested regional intermediate versus amyloid-positive effect or continuous CL association survived FDR correction.

In the independent model-native Z^{raw} intermediate versus amyloid-positive analysis, DAT-enriched right caudal middle frontal cortex again showed the strongest regional group effect. The pooled standardized effect was −0.698 (95% CI [−1.036, −0.361], $p < 0.001$, $q = 0.010$), with no evidence of between-batch heterogeneity ($I^2 = 0\%$). Batch-specific standardized effects were −0.689 in BBRC-ALFA, −0.630 in FACEHBI, and −0.758 in VUmc.

For subject-level RADB calculated from Z^{raw}, batch-stratified fixed-effect meta-analysis yielded a global

bioRxiv preprint doi: https://doi.org/10.64898/2026.09.28.754989; this version posted September 29, 2026. The copyright holder for this preprint (which was not certified by peer review) is the author/funder, who has granted bioRxiv a license to display the preprint in perpetuity. It is made available under a CC-BY 4.0 International license.

intermediate minus amyloid-positive standardized effect of 0.369 (95% CI 0.082–0.656, $p = 0.012$, $q = 0.047$), with $I^2 = 53.6\%$. System-specific Z^{raw} RADB effects did not survive the four-scope correction (DAT $q = 0.172$, NET $q = 0.060$, SERT $q = 0.054$). Using feature- and batch-specific empirical local-reference 95% limits for Z^{raw} extreme deviations, intermediate-burden minus amyloid-positive differences were 0.026 for global ($q = 0.044$), 0.034 for DAT ($q = 0.033$), 0.009 for NET ($q = 0.589$), and 0.036 for SERT ($q = 0.044$). Neither Z^{raw} regional nor subject-level continuous CL analyses yielded statistically significant associations after FDR corrections.

Fourteen scope-specific RADB values from nine participants were flagged by the 3×IQR outer-fence rule and were retained in the primary analyses. Excluding these values preserved positive intermediate minus amyloid-positive RADB coefficients across all four scopes, with no comparison surviving FDR correction (minimum $q = 0.141$). Natural-log transformation of RADB likewise yielded no significant group comparison after FDR correction (minimum $q = 0.117$).

The robustness of the regional intermediate amyloid burden versus amyloid-positive group coefficients to downstream covariate adjustment is illustrated in Supplementary Figure 8.

A.2.2 Robustness of the primary findings

The sensitivity analyses showed that the DAT-enriched right caudal middle frontal group difference was consistent in direction and magnitude across covariate specifications, score representations, and acquisition batches (batch-specific standardized effects −0.63 to −0.76; $I^2 = 0\%$). However, the corrected categorical findings were attenuated when the analysis was restricted to the most conservative 157-feature cross-batch diagnostic domain. Thus, the main regional finding was robust to downstream adjustment and deviation-score representation but remained sensitive to the definition of feature-level transfer quality. Independent replication with prospectively defined transfer criteria will be required to determine its generalizability.

bioRxiv preprint doi: https://doi.org/10.64898/2026.09.28.754989; this version posted September 29, 2026. The copyright holder for this preprint (which was not certified by peer review) is the author/funder, who has granted bioRxiv a license to display the preprint in perpetuity. It is made available under a CC-BY 4.0 International license.

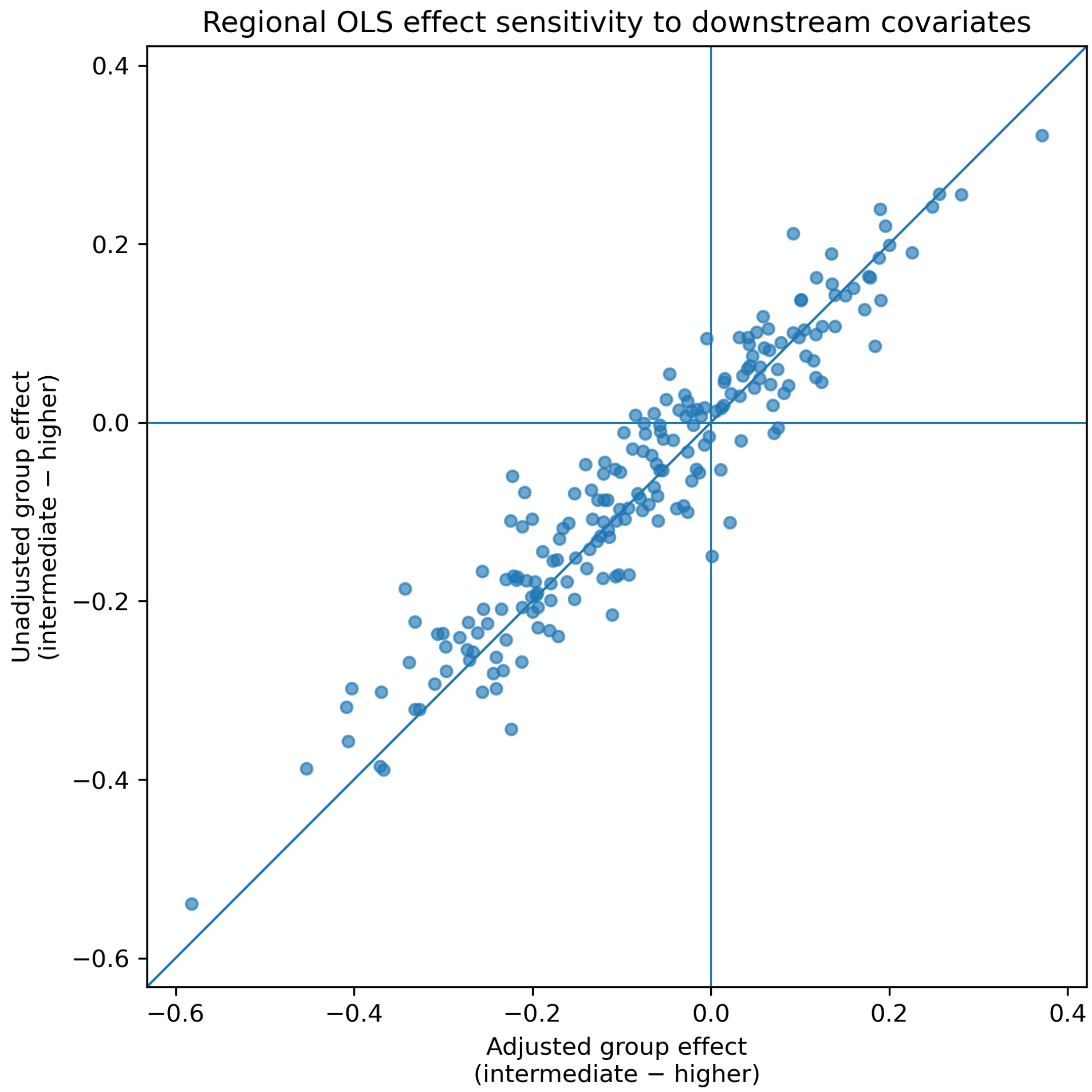

Figure 8: Sensitivity of regional intermediate minus amyloid-positive group coefficients to downstream covariate adjustment. Each point represents one cortical molecular-system feature. The horizontal axis shows the HC3 coefficient from the primary model adjusted for age, sex, and acquisition batch; the vertical axis shows the coefficient from the group-only HC3 model. The diagonal indicates equality of the two estimates.
